# miR-1202 acts as anti-oncomiR in myeloid leukaemia by down-modulating GATA-1_S_ expression

**DOI:** 10.1098/rsob.230319

**Published:** 2024-02-14

**Authors:** Raffaele Sessa, Silvia Trombetti, Alessandra Lo Bianco, Giovanni Amendola, Rosa Catapano, Elena Cesaro, Fara Petruzziello, Maria D'Armiento, Giuseppe Maria Maruotti, Giuseppe Menna, Paola Izzo, Michela Grosso

**Affiliations:** ^1^ Department of Molecular Medicine and Medical Biotechnology, University of Naples Federico II, Naples, Italy; ^2^ Department of Veterinary Medicine and Animal Productions, University of Naples Federico II, Naples, Italy; ^3^ Department of Public Health, Section of Pathology, University of Naples Federico II, Naples, Italy; ^4^ Gynecology and Obstetrics Unit, Department of Neuroscience, Reproductive Sciences and Dentistry, University of Naples Federico II, Naples, Italy; ^5^ Department of Pediatrics and Intensive Care Unit, Umberto I Hospital, Nocera Inferiore, Italy; ^6^ Department of Pediatric Hemato-Oncology, AORN Santobono-Pausilipon, Naples, Italy; ^7^ CEINGE-Biotecnologie Avanzate 'Franco Salvatore', Naples, Italy

**Keywords:** alternative splicing, Down syndrome, GATA-1, miR-1202, myeloid leukaemia

## Abstract

Transient abnormal myelopoiesis (TAM) is a Down syndrome-related pre-leukaemic condition characterized by somatic mutations in the haematopoietic transcription factor GATA-1 that result in exclusive production of its shorter isoform (GATA-1_S_). Given the common hallmark of altered miRNA expression profiles in haematological malignancies and the pro-leukaemic role of GATA-1_S_, we aimed to search for miRNAs potentially able to modulate the expression of GATA-1 isoforms. Starting from an *in silico* prediction of miRNA binding sites in the GATA-1 transcript, miR-1202 came into our sight as potential regulator of GATA-1 expression. Expression studies in K562 cells revealed that miR-1202 directly targets GATA-1, negatively regulates its expression, impairs GATA-1_S_ production, reduces cell proliferation, and increases apoptosis sensitivity. Furthermore, data from TAM and myeloid leukaemia patients provided substantial support to our study by showing that miR-1202 down-modulation is accompanied by increased GATA-1 levels, with more marked effects on GATA-1_S_. These findings indicate that miR-1202 acts as an anti-oncomiR in myeloid cells and may impact leukaemogenesis at least in part by down-modulating GATA-1_S_ levels.

## Graphical abstract

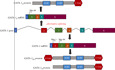

## 1. Introduction

**Figure 1.**
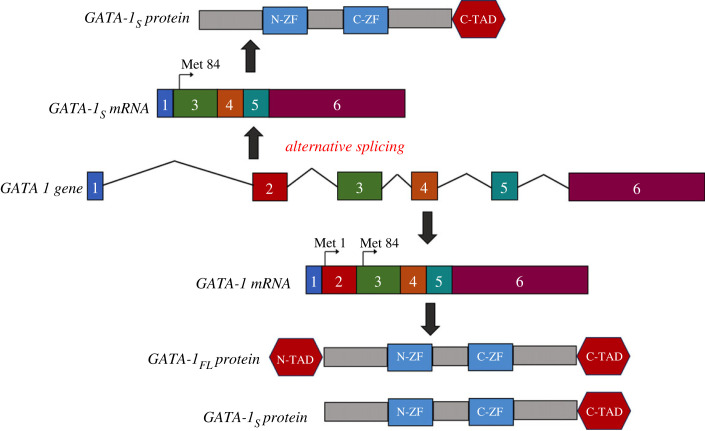
Schematic of post-transcriptional mechanisms generating GATA-1 isoforms.

The transcriptional factor GATA-1 plays a pivotal role in differentiation and maturation of several haematopoietic cell lineages. The gene *GATA-1* comprises six exons with one noncoding and five coding exons, and codifies for two protein isoforms: a full-length 413 amino acid protein (GATA-1_FL_) whose methionine initiation codon is located in exon 2 and which includes a N-terminal transactivation domain (exon 2-encoded TD), two conserved zinc finger domains encoded by exons 4 and 5 and a C-terminal domain, and a truncated 330 amino acid isoform lacking the N-terminal domain, referred to as GATA-1 short (GATA-1_S_). The shorter isoform can originate either from an alternatively spliced mRNA variant lacking exon 2 or from the use of an alternative translation initiation site (Met84) located in exon 3 (figure 1) [1–3]. These two isoforms have distinct roles in normal haematopoiesis: as the result of a complex interplay of transcriptional networks, GATA-1_FL_ promotes the terminal differentiation of megakaryocytic–erythroid lineages, whereas GATA-1_S_ enhances the proliferation and self-renewal of myeloid progenitors and contributes to the maintenance of the proliferative potency of haematopoietic precursors and skewing of the myeloid lineage [1–9]. Consequently, an imbalanced expression of GATA-1_S_ over GATA-1_FL_ has been identified as an oncogenic factor in several haematopoietic disorders including different subtypes of acute and chronic myeloid leukaemia [3,10–15]. According with the pro-leukaemic role exerted by GATA-1_S_, more recently we found that its dysregulated expression is associated with altered lipid metabolism and enhanced antioxidant activities, leading ferroptosis resistance to sustain proliferation programmes and survival pathways in normal or malignant haematopoiesis [16].

As a consequence of disturbed early haematopoietic differentiation patterns associated with trisomy 21 (T21), children with Down syndrome (DS) have a markedly high risk of developing leukaemia at infant age, particularly acute myeloid (AML-DS) and lymphoblastic leukaemia (ALL-DS), in striking contrast with the lower incidence of many solid tumours [17–20]. Myeloid leukaemia of DS (ML-DS) includes acute megakaryoblastic leukaemia (AMKL-DS) and myelodysplastic syndrome (MDS), a myeloproliferative disorder that often precedes AMKL-DS. These conditions originate from events that may occur in the prenatal and in the newborn periods [19]. Prenatally, T21 has a key role in priming transforming events in the haematopoietic microenvironment in the fetal liver that eventually lead to increased proliferation of megakaryocyte progenitors, thus representing the first hit predisposing to leukaemia [21,22]. However, although T21 is crucial for preleukaemia initiation, a second hit represented by *in utero* acquisition of somatic mutations of *GATA-1* is required for the onset of a preleukaemia condition, referred to as transient abnormal myelopoiesis (TAM), previously also known as DS-related transient myeloproliferative disorder (TMD-DS), that occurs in 5–10% of newborns with DS and is strictly dependent on the coexistence of T21 and GATA-1 mutations [22,23]. Clonal GATA-1 mutations reported in TAM include small insertions/deletions or point mutations clustered in exon 2 or at the beginning of exon 3 and prevent the translation of full-length GATA-1 thus resulting in exclusive production of GATA-1_S_ leading to thrombocytopenia and excess blasts (TAM blasts) in the peripheral blood [21,24]. Generally, TAM spontaneously resolves within the first months of life. However, in 20–30% of cases, the accumulation of karyotypic and molecular aberrations in a latent TAM clone will give rise to ML-DS within the first 2–4 years of life [14,20,25,26]. In fact, although GATA-1 mutations are a common hallmark of ML-DS cases and are critical for leukaemia progression, the development of ML-DS requires a third hit represented by the acquisition of additional molecular defects, predominantly in cohesin complex genes such as STAG2 [22,27].

To date, although it is well established that TAM is a disorder of fetal liver haematopoiesis, it remains mostly unknown which individual genetic elements on chromosome 21 (HSA21) are responsible for promoting leukaemia susceptibility in DS, as well as which molecular mechanisms underlie the cooperation between T21 and GATA-1_S_ expression [22]. In this context, based on the evidence that altered miRNA expression profiles are tumour-initiating events and a common hallmark of haematological malignancies [28], it has been hypothesized that HSA21complete or partial trisomy could promote over-expression of different miRNAs cooperating with GATA-1_S_ in the pathogenesis of AMKL. Interestingly, a link was found between a set of HSA21-encoded miRNAs (miR-99a, let-7c, miR-155, miR-125b-2) and leukaemogenesis [29–31]. Particularly, T21-dependent miR-125b-2 over-expression was found to have synergistic effects with GATA-1_S_ to promote proliferation and self-renewal of fetal myeloid cells. These findings indicate miR-125b-2 as a positive regulator of megakaryopoiesis and an oncomiR involved in the pathogenesis of TAM/AML-DS [32]. More recently, other microRNAs encoded by different chromosomes have been proposed as erythroid onco-miRNAs in ML-DS. Notably, among these, chromosome 8-encoded miR-486-5p resulted to be upregulated by GATA-1_S_ [33,34]. Prompted by this evidence, we recently aimed to investigate possible mechanisms underlying the link between T21, miRNAs and GATA-1_S_ in the development of TAM/AML-DS by searching for candidate miRNAs targeting GATA-1 and potentially involved in the aberrant expression of GATA-1_S_. To start exploring this hypothesis, we used a GATA-1 bait in a capture-and-cloning approach to interrogate small RNAs from human DS fetal liver samples that allowed us to identify miR-1202 as a miRNA candidate able to directly target GATA-1. This study also led us to identify a miR-1202 target sequence within the exon 2 of the GATA-1 transcript and to demonstrate that miR-1202 acts as anti-oncomiR in myeloid cells and may impact leukaemogenesis by down-modulating GATA-1_S_.

## 2. Results

### 2.1. Identification of miRNA candidates targeting the GATA-1 transcript

**Figure 2.**
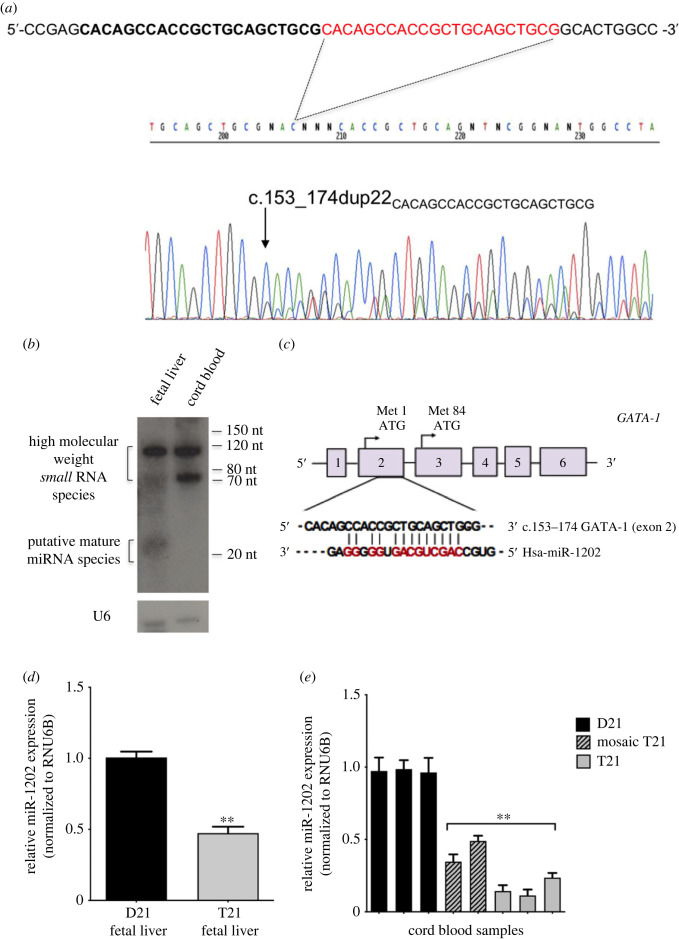
Identification of a putative target site of miR-1202 in the second exon of the GATA-1 transcript. (*a*) Sanger sequencing electropherogram of GATA-1 exon 2 showing a novel 22-nucleotide duplication in patient TAM-P1 (upper line, bold black and red text); the arrow indicates the starting point of the c.153_174 duplication sequence. (*b*) Northern blot analysis of small RNAs from fetal liver and cord blood samples. The upper panel is a representative image of hybridization signals obtained with an LNA probe corresponding to the 22-mer sequence (exon 2/22mer). Signals compatible with mature miRNA sizes were appreciable in the fetal liver sample. A probe against U6 RNA was used as loading control (lower panel). (*c*) *in silico* prediction of hsa-miR-1202 target sites showing pairing nucleotides (red text) between the miR-1202 seed region and its potential complementary site within the 22-mer sequence in the second exon of GATA-1. (*d*) Quantitative real-time PCR analysis in fetal liver samples showing reduced miR-1202 levels in a T21 sample as compared to a D21 control. (*e*) Quantitative real-time PCR analysis in cord blood samples showing reduced miR-1202 levels in mosaic or constitutional T21 samples as compared to D21 controls, with a more dramatic reduction in constitutional T21 samples. RNU6B was used as endogenous control. Data are presented as mean ± s.d. of three independent experiments. Statistics: *p-value < 0.05, **p-value < 0.001 relative to control groups (calculated by Student's *t*-tests and one-way ANOVA, followed by Dunnett's multiple comparisons test, where appropriate).

Characterization of *GATA-1* somatic mutations in TAM newborns led us to identify a tandem duplication of 22 nucleotides (c.153_174dup22CACAGCCACCGCTGCAGCTGCG) in the second exon of GATA-1 (patient TAM-P1, electronic supplementary material, table S1), a hitherto mutation that creates a frameshift and a premature termination codon, thus causing the exclusive production of GATA-1_S_ originated from the use of the alternative downstream translation start site in exon 3 (Met84) (figure 2*a*). Surprisingly, Blast analysis (http://blast.ncbi.nlm.nih.gov/Blast.cgi) revealed homology between the 22-mer duplicated sequence of GATA-1 exon 2 and a miRNAs cluster on chromosome 21. This finding prompted us to hypothesize a miRNA-mediated mechanism regulating GATA-1 isoforms expression.

To begin exploring this hypothesis, Northern blot analysis was performed using the 22-mer sequence (exon 2/22mer) as a probe to assess the existence of any hypothetical miRNA complementary to this GATA-1 region. For this analysis, small RNA samples (less than 200 nt) were extracted and purified from human HSA21 disomic (D21) cord blood and fetal liver samples, chosen as the main sources of haematopoietic precursors during fetal life. Data showed appreciable hybridization signals in the haematopoiesis-related fetal liver and cord blood compatible with the presence of small RNA molecules and capable of interacting with the exon 2/22mer GATA-1 probe sequence (figure 2*b*).

To isolate and identify potential candidate miRNAs targeting this GATA-1 region, we leveraged a capture-and-cloning approach on the fetal liver RNA extract according to evidence that TAM is a disorder originating during fetal liver haematopoiesis and encouraged by the detection of hybridization signals compatible with mature miRNA sizes (figure 2*b*) in this tissue sample.

This method is based on the use of a specific biotin-labelled oligonucleotide probe to capture complementary sequences from small RNA-enriched samples [35,36] (for more details, see electronic supplementary material, experimental procedures). To this aim, we designed a biotinylated exon2/22mer probe used as bait to capture miRNAs potentially able to target the second exon of GATA-1. Isolated complementary fragments were thus cloned and analysed by Sanger sequencing. Through this effort, three miRNAs, namely hsa-miR-29b2, hsa-miR-let7b2 and hsa-miR-1202, were identified by similarity search in the miRBase database (http://www.mirbase.org). Although none of them resulted to be encoded on chromosome 21, miRNA target prediction tools (miRwalK v.3.0, RNAhybrid v.2.2 and TargetScan v.7.2) [37–39] identified a potential target site of miR-1202 within the same 22-mer sequence of GATA-1 used as bait (figure 2*c*), whereas no pairing was found in the 3′-UTR of GATA-1. These findings further prompted us to investigate more deeply the potential role exerted by miR-1202 on GATA-1 expression.

### 2.2. miR-1202 is downregulated in constitutive and mosaic trisomy 21 haematopoietic tissues

Based on the results previously reported, we next examined the expression levels of hsa-miR-1202 in fetal haematopoietic tissues by quantitative RT-PCR analysis using small RNA samples extracted from human fetal liver and cord blood specimens. Quantitative real-time PCR analysis of T21 fetal liver samples showed a strong reduction (up to 50%) in miR-1202 levels as compared to D21 samples (figure 2*d*). Subsequently, small RNA samples were isolated from cord blood specimens of at term or near-term newborns with normal D21 or T21 (mosaic or constitutional) karyotypes for quantitative RT-PCR analysis. Results showed lower miR-1202 levels in both mosaic and constitutional T21 samples as compared to D21 controls with a more marked reduction in constitutional T21 newborns (figure 2*e*), thus suggesting that gene dosage alterations related to T21 inversely correlate with miR-1202 expression levels.

### 2.3. miR-1202 negatively regulates GATA-1 expression in the erythroleukaemia K562 cell line

**Figure 3.**
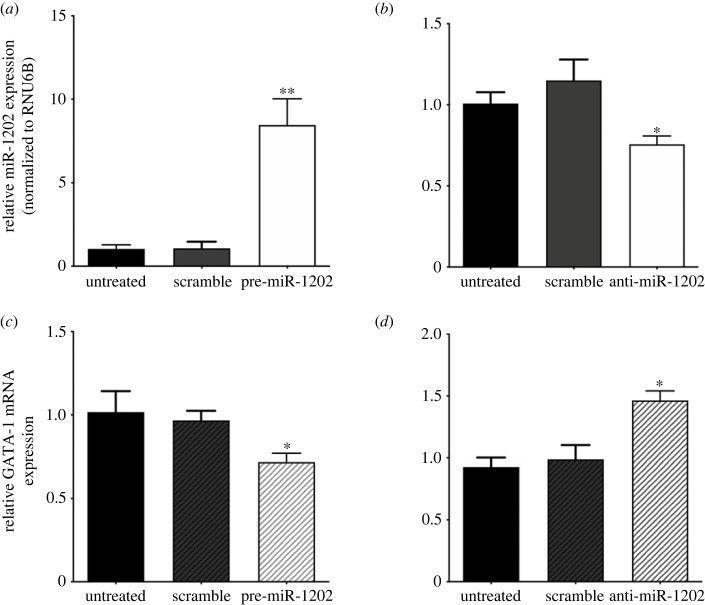
miR-1202 expression levels inversely correlate with GATA-1 mRNA levels in the myeloid leukaemia K562 cell line. (*a*,*b*) Evaluation of miR-1202 up- and downregulation in K562 cells transfected with (*a*) pre-miR-1202 or (*b*) anti-miR-1202, respectively. (*c*,*d*) endogenous GATA-1 mRNA levels in K562 cells transfected with (*c*) pre- and (*d*) anti-miR-1202, respectively. Data are presented as mean ± s.d. of three independent experiments. Statistics: *p-value < 0.05, **p-value < 0.001 versus untreated negative control groups (calculated by one-way ANOVA, followed by Dunnett's multiple comparisons test).

To better elucidate the potential role of miR-1202 on GATA-1 expression, we transiently transfected K562 cells with pre-miR-1202 or anti-miR-1202 oligomers and evaluated their effects on GATA-1 mRNA levels. Effectiveness of these treatments on up- and down-modulation of miR-1202 levels were verified by real-time PCR analysis on total GATA-1 mRNA levels (figure 3*a*,*b*). Interestingly, in these cells, miR-1202 upregulation was found associated with reduction of GATA-1 mRNA levels up to 30%, thus supporting the role of miR-1202 as a down-modulator of GATA-1 expression (figure 3*c*). Conversely, treatment with anti-miR-1202 resulted in increased GATA-1 production, providing further evidence that miR-1202 exerts a negative regulatory role on GATA-1 expression (figure 3*d*). As a whole, these findings are consistent with the hypothesis that miR-1202 could affect GATA-1 production almost in part by regulating mRNA stability.

### 2.4. miR-1202 differently affects the expression levels of GATA-1 isoforms

**Figure 4.**
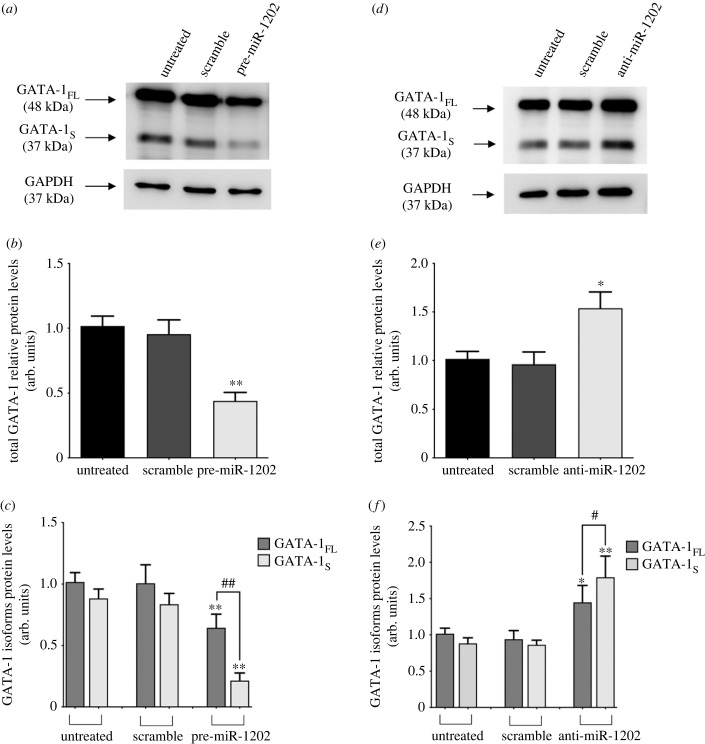
miR-1202 differently affects the expression levels of GATA-1 isoforms in K562 cells. (*a*) Western blot analysis of GATA-1 isoforms in protein extracts from K562 cells transfected with pre-miR-1202. GATA-1_FL_ is shown above with a protein band at approximately 48 kDa, with GATA-1_S_ represented by a band at 37 kDa. miR-1202 upregulation correlates with reduced endogenous GATA-1 levels, mostly due to the GATA-1_S_ fraction. (*b*,*c*) Densitometric analysis of western blot results showing (*b*) total GATA-1 levels, and (*c*) GATA-1 isoforms levels following miR-1202 up-regulation. (*d*) Western blot analysis of GATA-1 isoforms in protein extracts from K562 cells transfected with anti-miR-1202. miR-1202 downregulation was accompanied by increased levels of both GATA-1 isoforms with a significant prevalence of the GATA-1_S_ fraction. (*e*,*f*) Densitometric analysis of western blot results showing (*e*) total GATA-1 levels and (*f*) GATA-1 isoform levels. Data are presented as mean ± s.d. of three independent experiments. Statistics: *p-value < 0.05, **p-value < 0.001 versus untreated negative control groups and ^#^*p*-value < 0.05, ^##^*p*-value < 0.0001 versus each respective scramble control group (calculated by one-way ANOVA, followed by Dunnett's multiple comparisons test).

We next sought to verify that reduced miR-1202 levels would differently influence the expression of GATA-1 isoforms. To address this hypothesis, GATA-1 levels were evaluated by western blot assays performed on total protein extracts from K562 cells transiently transfected with pre- or anti-miR-1202 oligomers. To this aim, a GATA-1 antibody that is able to distinguish between the two GATA-1 isoforms was used. As shown in figure 4, miR-1202 upregulation in cells treated with pre-miR-1202 was accompanied by 50% reduction of total GATA-1 protein levels (figure 4*a*,*b*) with a more consistent effect on GATA-1_S_ levels (approx. 75% reduction) as compared to GATA-1_FL_ (approx. 30% reduction) (figure 4*c*). Conversely, down-modulation of miR-1202 in cells transfected with anti-miR-1202 resulted in increased total GATA-1 protein levels (figure 4*d*,*e*) with more marked effects on GATA-1_S_ (approx. 60% increase) as compared to GATA-1_FL_ (figure 4*f*). Collectively, besides providing further evidence that miR-1202 downregulates GATA-1 production (both at RNA and protein levels), these findings also indicate that miR-1202 contributes to regulate the GATA-1 isoforms ratio by exerting a more considerable inhibitory effect on the production of the pro-leukaemic GATA-1_S_ isoform.

### 2.5. Identification of GATA-1 as a direct target of miR-1202

**Figure 5.**
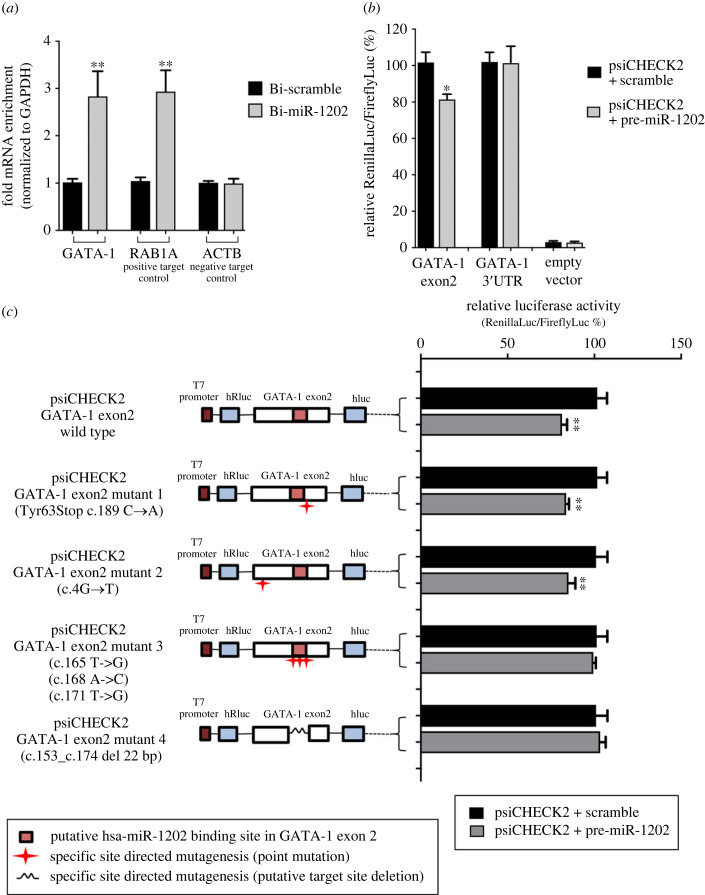
Identification of a miR-1202 binding element in the second exon of GATA-1. (*a*) Biotin-labelled miR-1202 (Bi-miR-1202) pull-down RNA samples showing a substantial enrichment (approx. threefold) in GATA-1 transcript by quantitative real-time PCR analysis. Enrichment analysis of RAB1A and ACTB transcripts were used as positive and negative controls, respectively. (*b*) Luciferase reporter gene assay in K562 cells co-transfected with pre-miR-1202 and GATA-1 exon 2 psiCHECK2 construct (GATA-1 exon 2) or GATA-1 3′-UTR psiCHECK2 construct. (*c*) Luciferase reporter gene assay in K562 cells co-transfected with wild-type or mutated GATA-1 exon 2 psiCHECK2 constructs. A reduced luciferase activity was found in cells transfected with the wild-type construct and the constructs bearing point mutations outside the putative miR-1202 binding site (mutant constructs 1–2). This effect was abolished by point mutations within the putative miR-1202 target site or its complete deletion (mutant constructs 3 and 4). Wild-type and mutant sequence details are shown in electronic supplementary material, table S1. Data are presented as mean ± s.d. of three independent experiments. Statistics: *p-value < 0.05, **p-value < 0.001 versus each respective scramble control group (calculated by one-way ANOVA, followed by Dunnett's multiple comparisons test).

We next questioned whether GATA-1 is a direct target of miR-1202. To this aim RNA pull-down experiments were conducted using a biotin-labelled miR-1202 mimics to isolate miR-1202-bound mRNAs, as previously reported [40]. A significant approximately threefold enrichment in GATA-1 mRNA was found in K562 cells transfected with miR-1202 mimics, thus demonstrating that miR-1202 is able to bind endogenous GATA-1 transcripts in a targeted manner (figure 5*a*). In this experiment, enrichment analysis of the Rab1A transcript, a well characterized miR-1202 target [41], and of the ACTB transcript lacking miR-1202 binding sites [42,43] were used as positive and negative controls, respectively, to validate the pull-down data (figure 5*a*). Using this approach, we were able to demonstrate a direct interaction between miR-1202 and the GATA-1 transcript, as initially postulated on the basis of the capture-and-cloning results.

Next, dual luciferase gene reporter assays were performed to verify the binding of miR-1202 to its putative target site within the second exon of GATA-1. To this aim, two recombinant vectors were generated containing GATA-1 3′-UTR (GATA-1_3′UTR_) or exon 2 (GATA-1_exon2_) sequences, respectively, cloned downstream of the Renilla luciferase reporter gene in psiCHECK-2 vector as previously described [44,45], specifically designed as biosensor for miRNA activity and a screening tool for miRNA targets [46]. These vectors were co-transfected in K562 cells with either a pre-miR-1202 or a scramble pre-miR control and the Renilla luciferase activity was evaluated and compared to the firefly luciferase activity. As shown in figure 5*b*, the dual luciferase gene reporter assay manifested an inhibitory effect of miR-1202 on the relative luciferase activity (approx. 20% reduction) in cells co-transfected with GATA-1_exon2_, thus further supporting the presence of a miR-1202 target sequence in the second exon of the GATA-1 transcript. Conversely, no significant variation in luciferase activity was detected in cells co-transfected with GATA-1_3′UTR_ and pre-miR-1202 as compared to the scramble control that were consistent with our previous *in silico* analysis indicating the lack of putative miR-1202 target sites in the 3′-UTR of the GATA-1 transcript.

Finally, to better define the miR-1202 putative binding site in GATA-1, we produced a set of mismatched GATA-1_exon2_ mutants including either point mutations or small deletions within or outside the miR-1202 putative target region (for more details, see electronic supplementary material, table S2). Interestingly, an inhibitory effect on the luciferase activity, comparable to the wild-type GATA-1_exon2_ vector, was detected only in cells co-transfected with constructs bearing mutations outside the putative miR-1202 target site. Conversely, mutations falling within the putative miRNA binding site led to unvaried luciferase activity with respect to the wild-type GATA-1_exon2_ vector, thus indicating that altering this string motif abolishes miR-1202-mediated repression of GATA-1 expression (figure 5*c*). As a whole, these results confirmed that miR-1202 targets the coding sequence (CDS) region of GATA-1 and allowed to define the direct binding site within its second exon and confirmed the lack of miR-1202 target sites in the 3′-UTR of GATA-1.

### 2.6. miR-1202 has an anti-proliferative and pro-apoptotic role in myeloid cells

**Figure 6.**
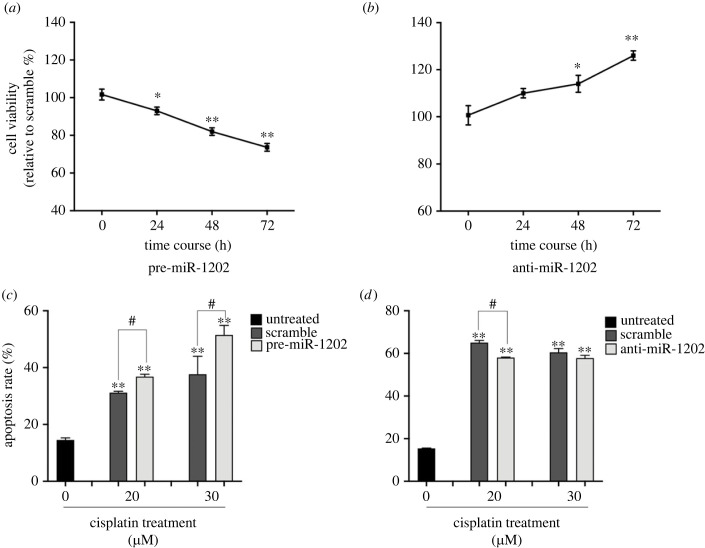
miR-1202 has anti-proliferative effects and increases apoptosis sensitivity in K562 cells. (*a*,*b*) Cell viability assessed by MTT assay on K562 cells 24, 48 and 72 h after transfection with (*a*) pre-miR-1202 or (*b*) anti-miR-1202. (*c*,*d*) Evaluation of apoptosis (Annexin V-FITC+/PI + cells) in K562 cells (*c*) upregulated or (*d*) down-modulated for miR-1202 and treated with 20 and 30 µM cisplatin. Data are presented as mean ± s.d. of three independent experiments. Statistics: (*a*) and (*b*) *p-value < 0.05, **p-value < 0.001 compared to the scramble control group (calculated as fold-change relative to control cells, arbitrarily set at 100%). (*c*) and (*d*) *p-value < 0.05, **p-value < 0.001 versus untreated K562 cells and ^#^*p*-value < 0.05, ^##^*p*-value < 0.0001 versus scramble control cells (calculated by one-way ANOVA, followed by Dunnett's multiple comparisons test).

**Figure 7.**
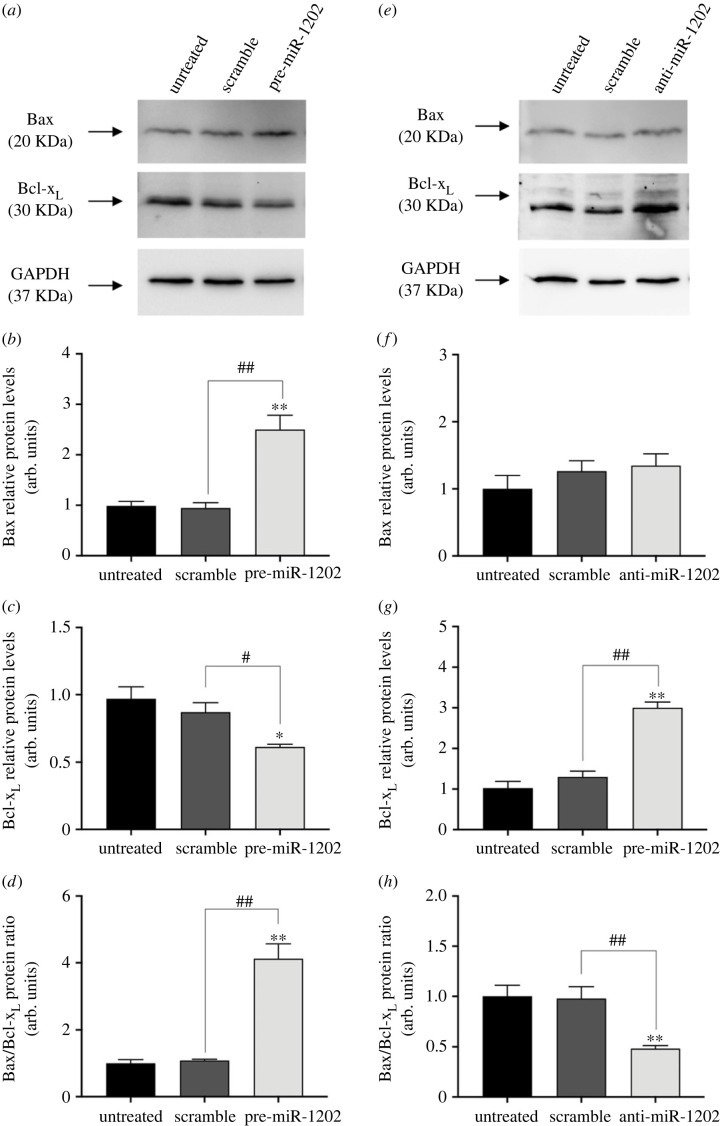
miR-1202 upregulation increases the apoptosis rate in K562 cells. (*a*) Increased expression of the pro-apoptotic protein Bax and decreased expression of the anti-apoptotic protein Bcl-x_L_ in K562 cells over-expressing miR-1202. (*b*–*d*) Densitometric analysis of western blot bands showing statistically significant (*b*) increased Bax levels (*c*) decreased Bcl-x_L_ levels and (*d*) increased Bax/Bcl-x_L_ expression ratio following miR-1202 up-modulation. (*e*) miR-1202 downregulation did not significantly affect the expression of Bax, but caused increased expression of Bcl-x_L_. (*f*–*h*) Densitometric analysis of western blot results showing (*f*) no significant variations in Bax levels, (*g*) significantly increased Bcl-x_L_ levels and (*h*) reduced Bax/Bcl-x_L_ expression ratio following miR-1202 down-modulation. For each analysis, band intensities from three independent experiments were quantified and normalized to GAPDH used as loading control. Data are presented as mean ± s.d. of three independent experiments. Statistics: *p-value < 0.05, **p-value < 0.001 versus untreated negative control groups and ^#^*p*-value < 0.05, ^##^*p*-value < 0.0001 versus scramble control groups (calculated by one-way ANOVA, followed by Dunnett's multiple comparisons test).

Since the expression of miR-1202 appears to exert significant inhibitory effects on the abundance of the pro-leukaemic GATA-1_S_ isoform, we thus hypothesized that miR-1202 could act as anti-oncomiR in myeloid leukaemia. To verify this hypothesis, we firstly performed MTT assays to evaluate the proliferative capacity of K562 cells treated with pre-miR-1202 or anti-miR-1202 oligomers. Results showed that cell proliferation ability was inhibited following miR-1202 over-expression, whereas the anti-miR-1202 treatment exerted the reverse effect (figure 6*a*,*b*). These results further supported the hypothesis on a potential onco-suppressor role of miR-1202 that may almost partly occur through the preferential targeting of the pro-proliferative GATA-1_S_ isoform as above described (§2.4). We investigated further whether the different proliferative phenotypes elicited upon over-expression or reduction of miR-1202 levels might be related to changes in apoptosis sensitivity. To this aim, K562 cells upregulated or down-modulated for miR-1202 were exposed to different doses of the pro-apoptotic drug cisplatin (20 and 30 µM). Results showed a significant dose-dependent increase of apoptotic rate following miR-1202 upregulation (figure 6*c*). By contrast, a reduction in apoptotic cells was detected after miR-1202 down-modulation (figure 6*d*). Based on these observations, we next examined the expression levels of the pro-apoptotic Bax and the anti-apoptotic Bcl-x_L_ proteins. Consistently with our hypothesis, over-expression of miR-1202 was accompanied by increased Bax levels (approx. 2.5-fold increase) and approx. 30% reduced Bcl-X_L_ levels with a significantly higher Bax/Bcl-x_L_ ratio (figure 7*a–d*). By contrast, cells treated with anti-miR-1202 showed an approximately threefold increase in Bcl-x_L_ levels with no significant variations in Bax levels that were responsible of a reduced Bax/Bcl-x_L_ ratio (figure 7*e–h*). Increased Bax/Bcl-x_L_ ratio is a critical factor regulating the activation of the intrinsic pathway of apoptosis and enhancing the cellular sensitivity to chemotherapeutic agents [47,48]. Therefore, the evidence of increased levels of the pro-apoptotic protein Bax and decreased levels of the anti-apoptotic protein Bcl-x_L_ elicited by miR-1202 upregulation are fully consistent with the pro-apoptotic role of miR-1202. Collectively, these findings allowed us to conclude that miR-1202 exerts anti-proliferative activity and enhances vulnerability to pro-apoptotic stimuli in myeloid cells.

### 2.7. miR-1202 levels inversely correlate with GATA-1 expression levels and disease progression in TAM- and AML-DS patients

Finally, to evaluate possible clinical implications to our findings in the context of T21-related haematopoietic disorders, we proceeded with the evaluation of miR-1202 levels in peripheral blood samples collected at diagnosis and during the follow-up of two TAM patients (TAM-P2 and TAM-P3). Both patients were positive for somatic mutations in the second exon of GATA-1, namely c.189C > A and c.4G > T, respectively (electronic supplementary material, table S1). Mutation analysis was performed by Sanger sequencing of PCR fragments generated with the primer sets as previously described [49]. A pool of total RNAs, obtained from peripheral blood samples of two T21 at term newborns with neither haematological disorders nor GATA-1 mutations was used as negative control (T21 negative control). This approach, besides confirming the correlation between T21 and low miR-1202 levels observed in T21 cord blood samples (figure 1*e*), also allowed us to highlight a relationship between miR-1202 levels and disease progression in these two patients.

**Figure 8.**
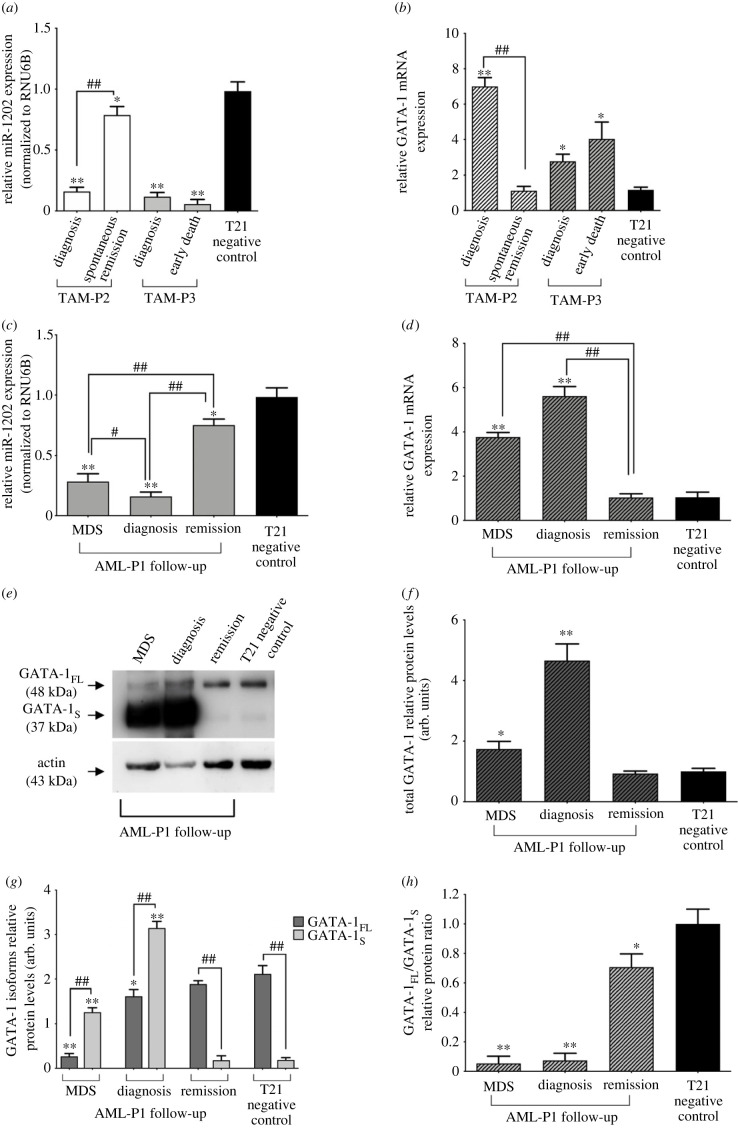
miR-1202 levels inversely correlate with GATA-1 expression levels in TAM and AML-DS patients. (*a*) miR-1202 mRNA levels detected in peripheral blood samples collected at different phases of disease in patients TAM-P2 and TAM-P3. (*b*) GATA-1 mRNA levels detected in patients TAM-P2 and TAM-P3 showing increased GATA-1 levels in both patients at diagnosis followed by GATA-1 downregulation at remission in patient TAM-P2, whereas further GATA-1 upregulation was detected in patient TAM-P3 with concomitant clinical worsening and early death. (*c*) miR-1202 mRNA levels in bone marrow samples from patient AML-P1 at different phases of disease. (*d*) An opposite trend was found in GATA-1 mRNA levels during MDS progression to AML showing over-expressed mRNA levels in the MDS phase that were further increased during AML progression and, conversely, dramatically reduced at complete remission. (*e*) Western blot analysis of GATA-1 isoforms in protein extracts from bone marrow samples of patient AML-P1 at different phases of disease. (*f*) Densitometric analysis of western blot results showing progressive increase of total GATA-1 levels during MDS to AML progression and its reduction to levels comparable to T21 control at remission. (*g*) Comparison of GATA-1 isoforms levels showing that both increased and reduced total GATA-1 levels were mostly due to the GATA-1_S_ fraction. (*h*) Evaluation of the GATA-1_FL_/GATA-1_S_ ratio confirming the altered ratio in favour of the GATA-1_S_ fraction during the acute phases of the disease and, conversely, an almost normalized ratio at remission. Band intensities were quantified and normalized to α-actin used as loading control. Statistics: *p-value < 0.05, **p-value < 0.001 versus each respective T21 negative control groups and ^#^*p*-value < 0.05, ^##^*p*-value < 0.0001 versus remission stage (calculated by one-way ANOVA, followed by Dunnett's multiple comparisons test).

Indeed, marked reduced levels of miR-1202 were initially found at TAM-DS diagnosis in both patients compared with the T21 negative control, opposite trends of miR-1202 expression levels were observed during their disease evolution. In fact, whereas TAM-P2 showed miR-1202 levels comparable to T21 negative control concomitantly with his complete remission, clinical worsening and fatal outcome in patient TAM-P3 were accompanied by further reduction of miR-1202 levels with respect to those detected at diagnosis (figure 8*a*).

In these two patients, quantitative real-time PCR analysis was also performed to evaluate GATA-1 transcript levels in order to verify a possible correlation with altered miR-1202 levels found in these samples. In both cases at diagnosis, reduced miR-1202 levels were accompanied by higher levels of the GATA-1 transcript as compared to T21 negative control. However, during the follow-up, GATA-1 mRNA levels were found decreased and almost comparable to T21 control in TAM-P2, concomitantly with increased miR-1202 levels and clinical remission, whereas maintenance of high GATA-1 levels in TAM-P3 correlated with persistent low miR-1202 levels and negative outcome (figure 8*b*). In summary, these data indicate an inverse correlation between miR-1202 and GATA-1 levels with higher miR-1202 levels being associated with lower GATA-1 expression and TAM remission; conversely, persistent down-modulated miR-1202 levels along with increased GATA-1 expression in TAM-P3 correlated with an adverse clinical outcome.

We also were able to evaluate miR-1202 and GATA-1 mRNA levels in bone marrow specimens from a patient with AML-DS (AML-P1) at different stages of disease (clinical and haematologic data listed in electronic supplementary material, table S1). miR-1202 relative expression levels were already significantly downregulated during the myelodysplastic phase (MDS) and further decreased concomitantly with disease progression in myeloid leukaemia (AML-DS) (figure 8*c*). Interestingly, even in this case, an opposite trend was observed in GATA-1 mRNA levels during the disease progression (figure 8*d*). Therefore, similarly to patient TAM-P2, an inverse relationship between miR-1202 and GATA-1 levels was found with increased miR-1202 levels and reduced GATA-1 positively correlating with disease remission.

We next verified that reduced miR-1202 levels in this patient would result in different expression of GATA-1 isoforms, as previously found in K562 cells. GATA-1 levels were evaluated by western blot assays performed on total protein extracts from AML-P1 bone marrow samples showing that elevated GATA-1 levels detected during the AML-DS progression were mostly due to the GATA-1_S_ fraction (figure 8*e*,*f*). Conversely, at remission, a dramatic reduction of GATA-1_S_ was found concomitantly with increased miR-1202 levels (figure 8*g*,*h*).

Collectively, data from TAM- and AML-DS patients were consistent with results obtained from K562 expression studies and further corroborated our findings.

## 3. Discussion

TAM is pre-leukaemic conditions occurring in DS newborns. Acquired mutations in the haematopoietic transcription factor GATA-1 are commonly detected in TAM blast cells leading to exclusive expression of the short isoform (GATA-1_S_), a protein variant lacking the N-terminal transactivation domain and originating from the use of an alternative translation initiation site (Met84) in exon 3 or from an alternatively spliced mRNA variant that omits exon 2. Indeed, two ATG start codons are used during the translation of the GATA-1 mRNA in erythroid cells. The first ATG is found in exon 2 and results in the full-length isoform, whereas the second ATG is found in exon 3 and results in the shorter isoform GATA-1_S_ [50,51].

Although GATA-1_S_ is known to play a critical role in the onset of TAM and subsequent ML development, the molecular mechanisms linking T21, acquired GATA-1 mutations and leukaemogenesis remain poorly understood.

miRNAs are widely involved in the regulation of processes such as apoptosis, cell differentiation and cell proliferation thus suppressing or promoting the progression of human cancers. In the context of leukaemogenesis, different miRNAs have emerged as important regulators of haematopoiesis [52–54]. Based on these observations, it has been proposed that dysregulated miRNA-mediated gene expression might cooperate with GATA-1_S_ in promoting pre-leukaemia in DS or contribute to control GATA-1 expression levels [21,24].

In an attempt to propose miRNA candidates that might contribute to explain this partially unexplored link, experimental data showing reduced miR-1202 levels in T21 fetal liver and cord blood samples along with *in silico* prediction of potential miR-target sites in the GATA-1 transcript initially provided us with a glimpse into a potential role played by hsa-miR-1202 in these processes. Existing literature also supported our research interest towards this miRNA. In fact, several lines of evidence are indicative of a link between miR1202, GATA-1 and leukaemia: (i) miR-1202 has been firstly isolated from leukaemia cells [55]; (ii) miR-1202 is encoded at locus 6q25.3, a cancer-associated genomic region involved in (6,21) translocation in acute myeloid leukaemia [56–58]; (iii) miR-1202 has a well-documented tumour suppressive role in different tumour tissues as it has been found downregulated in glioma, ovarian cancer and clear cell papillary renal carcinoma cells [41,59,60].

Herein we provide experimental evidence that miR-1202 negatively regulates GATA-1 expression and impairs GATA-1_S_ production by directly targeting its coding region. Indeed, a miR-1202 binding site was found in exon 2 of the GATA-1 transcript which serves to finely down-modulate the protein levels of GATA-1 with a more consistent effect on GATA-1_S_ as compared to GATA-1_FL_ (figure 4*c*). In the light of these findings, it is to be underlined that, besides the canonical mode of miRNA-mediated regulation of gene expression through 3′-UTR targeting, more recent lines of evidence indicate that other miRNA-dependent mechanisms may contribute to regulate mRNAs translation or stabilization in mammalian cells including miRNA targeting to CDSs thus providing further insights into the sophisticated regulation of many processes such as the modulation of the relative abundance of alternative spliced variants [61–68]. Consistently with these observations, in our study we found that upregulation of miR-1202 was accompanied by reduced levels of GATA-1 both at mRNA and protein levels along with more enhanced reduction of GATA-1_S_.

**Figure 9.**
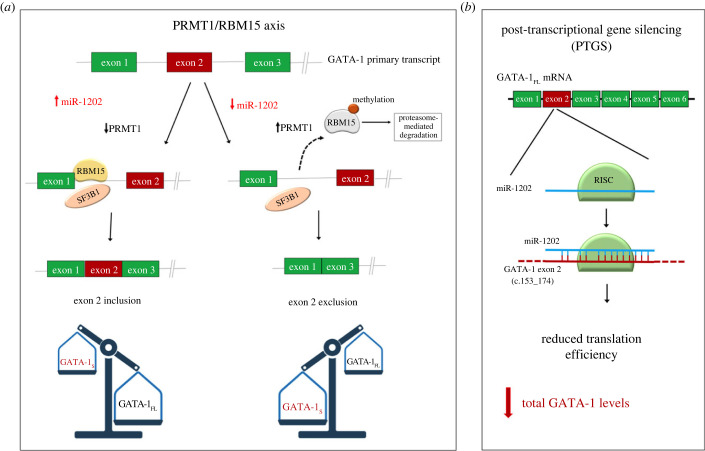
Schematic of putative molecular mechanisms involved in miRNA-mediated modulation of GATA-1 levels. (*a*) Regulation of alternative splicing of the GATA-1 transcript by the PRMT1/RBM15 axis. miR-1202 downregulation could promote PRMT1 activity, thus favouring alternative splicing of the GATA-1 transcript and production of the shorter isoform GATA-1_S_. (*b*) miR-1202 targets the coding region of the GATA-1 transcript to down-modulate GATA-1 levels through a post-transcriptional gene silencing mechanism (PTGS).

In this context, an interesting study provided a glimpse into the involvement of the PRMT1–RBM15 axis in the regulation of GATA-1 alternative splicing [69]. RBM15 is an RNA binding protein that binds to pre-messenger RNA intronic regions of genes involved in haematopoiesis such as GATA-1, RUNX1 and TAL1 and controls mRNA splicing to produce full-length transcripts through the recruitment of the splicing factor SF3B1 (figure 9) [70,71]. In this study, reduced RBM15 protein levels were found to promote the accumulation of GATA-1_S_, thus lowering the GATA-1_FL_/GATA-1_S_ ratio, whereas over-expression of RBM15 reversed the GATA-1_FL_/GATA-1_S_ ratio in favour of the full-length isoform. RBM15 methylation by protein arginine methyltransferase 1 (PRMT1) leads to its degradation through an ubiquitylation-mediated pathway [69]. Therefore, it has been assumed that PRMT1 promotes alternative RNA splicing of these transcripts by reducing RBM15 protein levels. Noteworthy, PRMT1 has been recently found highly expressed in acute myeloid and lymphoid leukaemia [72,73]. It is thus arguable that increased levels of PRMT1 may trigger alternative splicing processes leading to enhanced GATA-1_S_ production and unbalanced GATA-1_FL_/GATA-1_S_ ratio. Preliminary data recently obtained in our laboratory indicated an inverse correlation between miR-1202 and PRMT1 expression levels, hence suggesting a possible mechanistic explanation to the link between miR-1202 and GATA-1_S_ production. In the light of these findings, although further research is required to clarify the processes regulating the expression of GATA-1 and its isoforms, we might thus speculate that miR-1202 down-modulation could promote PRMT1 activity, thus stimulating alternative splicing processes and pushing toward the production of the pro-leukaemia isoform GATA-1_S_ (figure 9). Therefore, to summarize, our study demonstrates that miR-1202 down-modulates GATA-1_S_ production by contributing to two distinct but not mutually exclusive mechanisms: fine regulation of the mRNA stability and translational efficiency by direct binding to exon 2 which affects both GATA-1 isoforms albeit with more emphasis on the GATA-1_S_ output (figure 4*c*) and indirectly inhibition of exon 2 alternative splicing which exclusively affects GATA-1_S_ production. Anyway, based on these complex and unusual mechanisms of gene expression, it is not possible to distinguish the translated GATA-1_S_ product originated from the two different GATA-1 transcripts.

Thus, although several questions remain to be answered, the data herein presented lead us to hypothesize that a complex combination of splicing, translation and mRNA stability processes almost in part mediated by miR-1202 may be into place to fine-tune GATA-1 expression and to elegantly control the GATA-1_FL_/GATA-1_S_ ratio.

This study also led us to prove that miR-1202 inhibits proliferation and sensitizes myeloid cells to apoptosis thus acting as an anti-oncomiR. As regards the role of miR-1202 in T21-dependent myeloproliferative disorders, data from TAM and myeloid leukaemia patients provided substantial support to our findings by showing that miR-1202 down-modulation at diagnosis is accompanied by elevated GATA-1 levels, with more marked effects on GATA-1_S_. As a whole, our results suggest that miR-1202 down-modulation may be an early event, required for the origin and maintenance of pre-leukaemia clones. Subsequent acquisition of somatic GATA-1 mutations may confer further enhanced proliferative and anti-apoptotic capacities to TAM blasts. Although the putative mechanistic link between T21 and miR-1202 down-modulation is yet to be deciphered, evidence of increased miR-1202 levels in MDS patients treated with azacitidine, an hypo-methylation agent, is suggestive of T21-dependent epigenetic processes leading to impaired miR-1202 expression [74–76].

In conclusion, besides contributing to expand our knowledge on the molecular mechanisms underlying miRNA regulatory functions, our study provides the first proof-of-concept for miR-1202 downregulation as a factor promoting the expression of the pro-leukaemic factor GATA-1_S_. Our demonstration of miR-1202 acting as an anti-oncomiR in myeloid cells along with its reduced expression in T21-haematopoietic tissues extends our understanding of leukaemia susceptibility in DS and could provide the missing link in the cooperative effects of T21 and acquisition of somatic GATA-1 mutations in TAM onset and AML-DS development. Although further investigations are required, our study sheds novel light on the very early steps of the leukaemogenic process and raises the possibility of enhancing miR-1202 expression as a potential therapeutic intervention in myeloid leukaemia.

## 4. Material and methods

### 4.1. Patients and specimens

Residual peripheral blood samples or bone marrow specimens collected at diagnosis and at different phases of disease from three TAM-DS newborns and an AML-DS patient were used in this study (molecular and clinical details are shown in electronic supplementary material, table S1). Eight cord blood samples from D21, mosaic or constitutional T21 at term or near-term newborns and fetal liver samples of D21 and T21 fetuses (16–23 weeks of gestation) were obtained after elective pregnancy termination. DS patients with no haematological disease or *GATA-1* mutations (T21 negative control) were included in the study.

The study design was approved by our local Institutional Research Ethics Committee (project approval number 462/21). The study was conducted according to the criteria set by the declaration of Helsinki and the Belmont Report. Informed consent was obtained from individuals or their guardians for all samples used in this study.

### 4.2. Cell culture

The human erythroleukaemia K562 cells from European Collection of Authenticated Cell Culture (ECACC, no. 89122407) were cultured in RPMI 1640 medium (Sigma-Aldrich, St. Louis, MO, USA, no. R0883) supplemented with 10% fetal bovine serum (FBS) (Sigma-Aldrich, no. F7524) plus 2 mM glutamax (Gibco, Thermo Fisher Scientific Inc., Waltham, MA, USA, no. 35050061) in 25 cm^2^ tissue culture flask. Cells were incubated at 37°C in a humidified 5% CO_2_-containing atmosphere and were kept at 70–80% confluence for transient transfection experiments. K562 were free of mycoplasma, authenticated on the basis of morphology and growth properties and confirmed by PCR mycoplasma test kit (AppliChem, Darmstadt, Germany, no. A3744).

### 4.3. Cell transfection

K562 cells were transiently transfected after 7-day culture with Lipofectamine 2000 Reagent (Invitrogen, Thermo Fisher Scientific, no. 11668019), according to the manufacturer's instructions. Two hours before transfection, cells were seeded into 6-well plates at a density of 5 × 10^5^ in 2 ml of RPMI 1640 medium without serum. K562 were transiently transfected with the miRNA precursor pre-miR-1202 (Life Technologies, Thermo Fisher Scientific, no. PM13442) or miRNA antagonist anti-miR-1202 (Life Technologies, no. AM13442) and pre-miR Negative Control no. 1 (Life Technologies, no. AM17121) or anti-miR Negative Control no. 1 (Life Technologies, no. AM17012). All miRNA precursors for transfections were used at a final concentration of 45 nM, following evaluation of optimal experimental conditions. Six hours after transfection, growth medium was supplemented with 10% FBS. Forty-eight hours after transfection, cells were harvested for total RNA and protein extraction for further studies.

### 4.4. RNA analysis

#### 4.4.1. Total and small RNA extraction

Total and small RNAs were extracted from cell lines, fetal liver and blood samples with the Qiazol reagent (Qiagen GmbH, Hilden, Germany) and mirVana miRNA Isolation kit (Invitrogen, no. AM1560), respectively, according to the procedures recommended by the manufacturers. RNA was quantized spectrophotometrically, DNA contamination was excluded by gel electrophoresis on a 1.5% denaturing agarose gel in MOPS 1× buffer (20 mM MOPS pH 7.0, 8 mM Sodium Acetate, 1 mM EDTA pH 8.0).

#### 4.4.2. Quantitative real-time PCR analysis

cDNA was synthesized from 250 ng of total RNA using the QuantiTect Reverse Transcription Kit (Qiagen, no. 205310) according to manufacturer's protocols. RT-PCR procedures were performed on a CFX96 Real-Time System (Bio-Rad Laboratories, Hercules, CA, USA). The primer set used for GATA-1 and GAPDH (endogenous control) transcripts are shown in electronic supplementary material, table S3. In details, GATA-1 primers were designed in order to amplify a common region (exons 4–6) to both regular and alternatively spliced GATA-1 transcripts. Each real-time PCR was performed in triplicate in a 20 µl reaction mix containing 10 µl of 2× Sso Advanced Universal SYBR Green Supermix (Bio-Rad Laboratories), 0.38 µl of a 20 µM primer mix, 2 µl of cDNA (1/10 volume of RT-PCR product) and 7.62 µl of nuclease-free water. The cycling conditions were set up as follows: an initial denaturation step at 95°C for 3 min, followed by 40 cycles (95°C for 15 s, 60°C for 30 s) and 80 cycles, according to standard protocols for melting curve analysis. The calibration curve for assessing the efficiency of the PCR reaction was performed on at least three serial dilutions (1 : 10) of the reverse transcriptase products.

MicroRNAs were reverse transcribed using the TaqMan microRNA Reverse Transcription Kit (Applied Biosystems, Life Technologies, no. 4366597) and amplified by TaqMan Universal Master Mix II (Applied Biosystems, Life Technologies, no. 4440040) according to the manufacturer's instructions. Relative expression levels were calculated using miRNA TaqMan probes for hsa-miR-1202 (Life Technologies, no. 002858) and RNU6B, used as the endogenous control (Applied Biosystems, Life Technologies, no. 001093). Real-time PCR reactions were run in triplicates using the CFX96 Real-Time System (Bio-Rad Laboratories) and CT values were determined by automated threshold analysis. Data were analysed using the CFX Manager 3.0 software (Bio-Rad Laboratories) according to the manufacturer's specifications.

### 4.5. Western blot analysis

Total protein extraction from K562 cells was performed as previously described [13]. Protein extraction from blood and bone marrow specimens was performed using the Qiazol (Qiagen) procedure according to the manufacturer's instructions. Western blots analysis was performed as previously reported [77,78]. Primary and secondary antibodies are listed in electronic supplementary material, table S4. Quantitative densitometry of bands was carried out by analysing ChemiDoc XRS Image System (Bio-Rad Laboratories), and the quantification of the signal was performed by ImageJ software.

### 4.6. Cell proliferation assay

The MTT [3-(4,5-dimethyl-2-tetrazolyl)-2,5-diphenyl-2H tetrazolium bromide] Cell Proliferation assay (Cell Proliferation Kit 1, Roche, Basilea, Switzerland) measures the cell proliferation rate and conversely, when metabolic events lead to apoptosis or necrosis, the reduction in cell viability. K562 cells were transfected using Lipofectamine 2000 as transfectant agent with 45 nM of Pre-miR-1202 and/or anti-miR-1202, respectively, for 24 h, 48 h and 72 h. Pre-miR and anti-miR scrambles were used at the same concentrations as negative controls. After transient transfection, K562 cells were seeded into a 96-well plate at a concentration of 1.5 × 10^4^ cells 100 µl. After treatments, 10 µl of MTT labelling reagent were added to each well according to the manufacturer's instructions. MTT, a yellow tetrazole, is reduced to purple formazan in living cells. Ten microlitres of solubilization buffer 1× (10% SDS in 0.01 M HCl) was added to dissolve the insoluble purple formazan product into a coloured solution. Measurement of the soluble formazan product in each well was measured by photometric reading at 570/690 nm on a Synergy H1 Hybrid Multi-Mode Microplate Reader (BioTek, Winooski, VT, USA). The degree of light absorption is dependent on the formazan concentration accumulated inside the cell and on the cell surface. The greater the formazan concentration, the deeper is the purple dye and thus lower the absorbance rate. Viability data were calculated as fold-change relative to scramble transfected cells (arbitrarily set at 100%) and plotted on graphs as mean ± s.d. of three independent experiments.

### 4.7. Cell apoptosis

Forty-eight hours after transfection with pre- or anti-miR-1202 oligomers, K562 cells were treated for 16 h with 20 and 30 µM cisplatin (Sigma-Aldrich, no. 232120). Briefly, after cisplatin exposure, cells were harvested, washed in PBS 1×, and resuspended in 1× Annexin binding buffer (10 mM Hepes/NaOH (pH 7.4), 0.14 M NaCl, 2.5 mM CaCl_2_) at a concentration of 1 × 10^6^ cells ml^−1^. Following the manufacturer's protocol for the Annexin V-FITC Apoptosis detection Kit I (BD Biosciences, San Diego, CA, USA, no. 556547), 100 µl of cell suspension were double stained with Annexin V-FITC and propidium iodide (PI) for 10 min in the dark. Then, 400 µl of 1× Annexin binding buffer were added to each sample and analysed by flow cytometry using an Accuri C6 flow cytometer and BD Accuri C-Flow software (BD Biosciences) [79].

### 4.8. RNA pull-down assay

K562 cells were transiently transfected with Lipofectamine 2000 Reagent into six-well plates at a density of 5 × 10^5^ in 2 ml of serum-free RPMI 1640 medium in a mixture containing 20 nM of 3′-biotin-labelled miR-1202 mimic (Dharmacon, Lafayette, CO, USA, no. CTM483347) or 3′-biotin-labelled Cel-miR-67 mimic (Bi-CTL) used as negative control (Dharmacon, no. CTM-483343). Six hours after transfection, the medium was supplemented with 10% FBS in each well and cells were harvested 48 h after transfection. Approximately 1 × 10^5^ K562 cells were collected and added with 600 µl pull-down buffer (Tris HCl 20 mM pH 7.5, KCl 100 mM, 5 mM MgCl_2_, 0.3% IGEPAL CA-630, 1× mixture Protein inhibitor Cocktail-Sigma-Aldrich and 5 U ml^−1^ of RNaseOUT) and incubated for 4 h with 50 µl pre-coated streptavidin-Dyna beads M-280 (Invitrogen) at 4°C with gentle rotation. Pull-down and input RNA samples were extracted and analysed by quantitative real-time PCR. GAPDH was used as internal control to normalize the qRT-PCR results. Data analyses were performed by calculating the miRNA enrichment as follows:

Bi-miR-1202 pull-down for GATA-1 mRNA/scramble control pull-down for GATA-1 mRNA = X

Bi-miR-1202 input/scramble control input = Y; Fold binding = X/Y

Results are expressed as the means ± s.d. from three independent experiments with three replicates for each set of experiments.

The β-actin transcript (ACTB, NM_001101.5) was used as negative control, whereas enrichment of Rab1a mRNA (RAB1A, NM_004161.5), a member of RAS superfamily of GTPases already described as a miR-1202 target [41] was used as positive control.

### 4.9. Dual luciferase reporter assay

PsiCHECK-2 vectors (Promega, no. C8021), originally designed to evaluate RNA interference (RNAi) efficiency, are used as screening tool for miRNA targets and biosensors for miRNA activity through rapid detection of expression changes of a target gene (Renilla luciferase gene) fused to a reporter gene (Firefly luciferase gene). The region of interest is cloned into a multiple cloning region located downstream of the Renilla translational stop codon. Two recombinant constructs were obtained containing GATA-1 3′-UTR (GATA-1_3′UTR_) or exon 2 (GATA-1_exon2_) sequences, respectively, cloned downstream of the Renilla reporter gene in the XhoI/NotI restriction sites of the multiple cloning region as previously reported [44,45].

K562 were seeded into 6 well plates at a density of 5 × 10^5^ in 2 ml of RPMI 1640 medium (Gibco) without FBS. K562 were transiently co-transfected using Lipofectamine 2000 as transfectant agent, in a mixture containing 45 nM of pre-miR-1202 miRNA precursor (assay ID PM13442 Life Technologies) and 300 ng of GATA-1_3′UTR_ or GATA-1_exon2_ vectors. Cells were collected 48 h after the transfection and analysed with the Dual-Glo Luciferase Assay Kit (Promega, no. E2920) according to manufacturer's instructions [80]. All experiments were performed in triplicates and repeated three times. Renilla (Rluc) and Firefly (Fluc) luciferase chemioluminescent signals were measured using a 20/20n luminometer (Turner Biosystems, Sunnyvale, CA, USA). To better define the putative hsa-miR-1202 seed sequence, mutant constructs were produced with the QuickChange II Site-directed mutagenesis kit (Agilent, Santa Clara, CA, USA, no. 200523) to generate point mutations and deletions within or outside the putative miR-1202 binding site in the GATA-1 exon2 psiCHECK-2 Vector (electronic supplementary material, table S2). To account for nonspecific effects on reporter plasmids, the relative ratio Rluc/Fluc of our experimental samples was further normalized on the background ratio Rluc/Fluc obtained using psiCHECK-2 empty vector cotrasfected with either scramble or pre-miR-1202.

### 4.10. Statistical analysis

All statistics were analysed with GraphPad Prism 8.0 (GraphPad Software, San Diego, CA, USA). Data were expressed as mean ± standard deviation (s.d.) and Student's *t*-tests were performed to analyse the data with normal distribution between two groups. Statistical analysis was performed using one-way ANOVA, followed by Dunnett's multiple comparisons test (*post hoc* test) for more than 2 experimental groups. All experiments were repeated in triplicate for at least three times. Differences versus each respective negative control were considered significant at *p < 0.05, **p < 0.0001 and ^#^*p* < 0.05, ^##^*p* < 0.0001 versus scramble control groups.

## Data Availability

All data generated or analysed during this study are included in this published article and its electronic supplementary material [81].
